# Allylic alcohols and amines by carbenoid eliminative cross-coupling using epoxides or aziridines

**DOI:** 10.3762/bjoc.17.155

**Published:** 2021-09-10

**Authors:** Matthew J Fleming, David M Hodgson

**Affiliations:** 1 Department of Chemistry, Chemistry Research Laboratory, University of Oxford, Mansfield Road, Oxford OX1 3TA, United Kingdom

**Keywords:** alkenes, aziridines, epoxides, lithiation, synthetic methods

## Abstract

α-Lithiated terminal epoxides and *N*-(*tert*-butylsulfonyl)aziridines undergo eliminative cross-coupling with α-lithio ethers, to give convergent access to allylic alcohols and allylic amines, respectively. The process can be considered as proceeding by selective strain-relieving attack (ring-opening) of the lithiated three-membered heterocycle by the lithio ether and then selective β-elimination of lithium alkoxide.

## Introduction

**Scheme 1:**
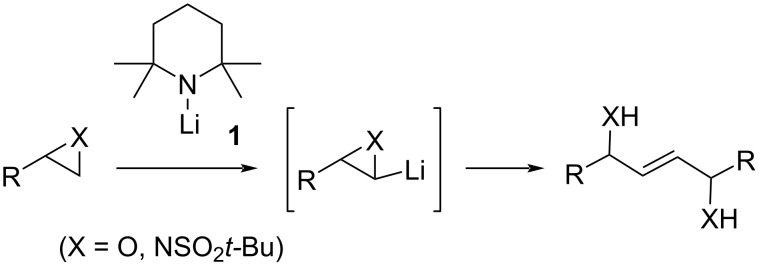
Dimerisation of α-lithio epoxides or aziridines [3–5].

**Scheme 2:**
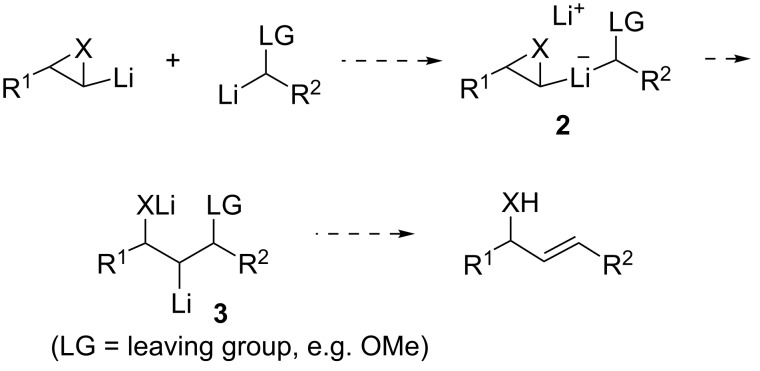
Proposed eliminative cross-coupling of carbenoids to allylic alcohols (X = O) or allylic amines (X = NSO_2_*t-*Bu).

Methods for the convergent generation of alkenes can be of significant utility in organic synthesis [1]. A relatively under-examined approach is through the interaction of two carbenoids [2]. Dimerisation of carbenoids may compete with a desired carbenoid transformation although its value has been demonstrated in, for example, our studies on lithium 2,2,6,6-tetramethylpiperidide (**1**, LTMP)-induced syntheses of 2-ene-1,4-diols and 2-ene-1,4-diamines from terminal epoxides [3] and aziridines [4–5], respectively (Scheme 1). The eliminative cross-coupling of carbenoids can provide a way to unsymmetrical alkenes, provided the differential reactivity of the two carbenoids is suitably matched [2]. In the current letter, we report preliminary results on the latter strategy to form alkenes which possess an allylic heteroatom (hydroxy, amino) functionality (Scheme 2).

## Results and Discussion

**Scheme 3:**
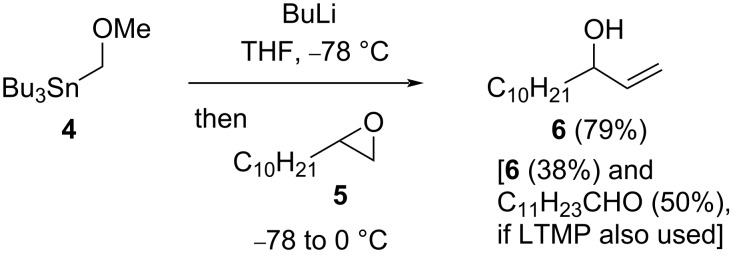
Allylic alcohol **6** by one-carbon homologation from epoxide **5**.

Our studies began (Scheme 3) by reaction of BuLi (4 equiv) with a mixture of stannane **4** [6] (2 equiv) and tetramethylpiperidine (TMP, 2 equiv), to generate methoxymethyllithium and LTMP, followed by addition of terminal epoxide **5**. This led to the desired allylic alcohol **6** (38%), likely via the selective (ring strain-relieving) 1,2-metalate rearrangement outlined in Scheme 2 (**2→3**, X = O, LG = OMe), then preferential β-elimination [7–8] of lithium methoxide rather than dilithium oxide. However, also isolated was dodecanal (50%), which arises from hydrolysis during work-up of the enamine that is formed from trapping of the lithiated epoxide by LTMP [9–10]. Omitting LTMP gave a significantly improved yield of the allylic alcohol **6** (79%, using BuLi and stannane **4** (3 equiv each)). This latter result suggests that methoxymethyllithium is capable of deprotonating terminal epoxide **5**, and this occurs in preference to direct attack at the (unlithiated) epoxide **5**. In contrast, no reaction was observed with a 2,2-disubstituted epoxide: 1-oxaspiro[2.11]tetradecane (**9**) [11] being recovered (90%) under the reaction conditions.

The one-carbon homologation of an epoxide to an allylic alcohol (cf Scheme 3) can also be achieved using excess dimethylsulfonium methylide [12–13], although non-terminal alkenes have not been shown to be directly accessible by higher homologation. To examine the latter in the context of the current chemistry, α-methoxyhexyllithium derived from stannane **7** [14–15] was reacted with terminal epoxide **5**, which gave the allylic alcohol **8** (79%, *E*/*Z* = 73:27, Scheme 4). This organolithium also proved reactive with 2,2-disubstituted epoxide **9**, giving allylic tertiary alcohol **10** (72%, *E*/*Z* = 82:18).

**Scheme 4:**
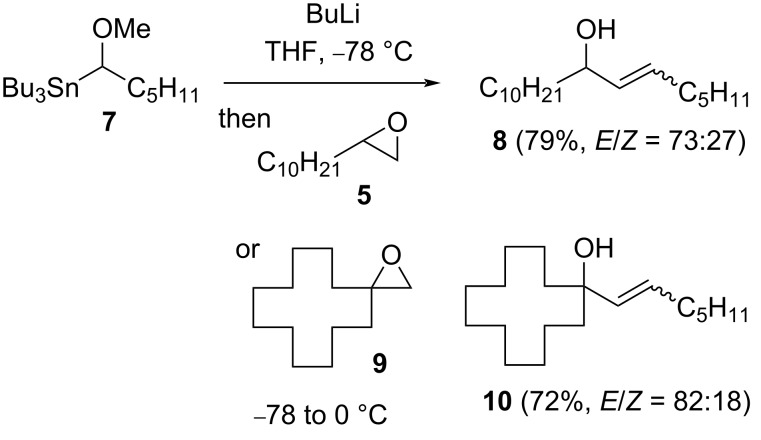
Internal allylic alcohols from epoxides and stannane **7**.

**Scheme 5:**
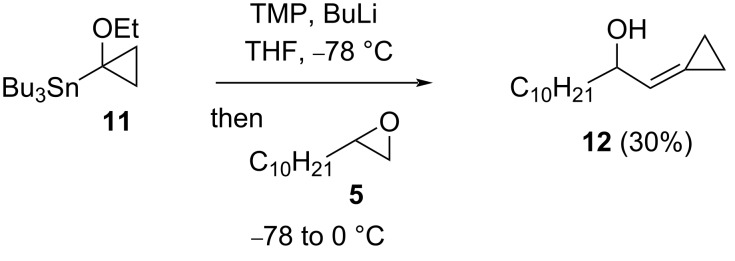
Cyclopropylidene synthesis from epoxide **5**.

A trisubstituted alkene **12** (30%) could be formed from terminal epoxide **5**, using cyclopropylstannane **11** [16] (Scheme 5); in this case the presence of LTMP was also necessary as epoxide **5** was recovered (>80%) in its absence.

**Scheme 6:**
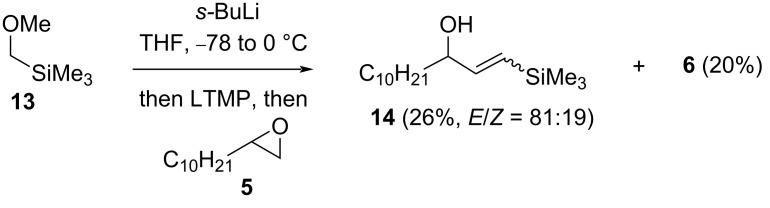
Synthesis of vinylsilane **14**.

A silyl-stabilised methoxymethyllithium, available by direct lithiation of (methoxymethyl)trimethylsilane (**13**) [17], gave vinylsilane **14** (26%, *E*/*Z* = 81:19) on reaction with terminal epoxide **5** in the presence of LTMP (Scheme 6); the allylic alcohol **6** was also isolated (20%), suggesting that in our hands lithium–trimethylsilyl exchange competes with lithiation of (methoxymethyl)trimethylsilane (**13**).

**Scheme 7:**
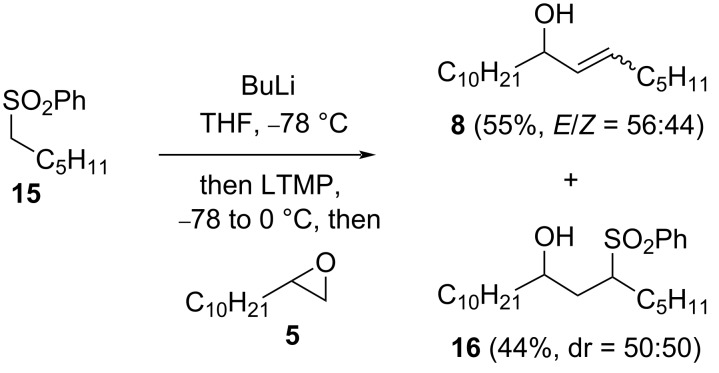
Allylic alcohol **8** from epoxide **5** and sulfone **15**.

Access to allylic alcohol **8** was also achievable (55%, *E*/*Z* = 56:44) in a tin-free process using a sulfonyl leaving group, via α-lithiation of sulfone **15** [18] and in the presence of LTMP (Scheme 7). γ-Hydroxysulfone **16** was formed competitively (44%, dr = 50:50), by direct addition of the lithiated sulfone to (unlithiated) epoxide **5** and was formed quantitatively (dr = 57:43) if the LTMP was omitted.

**Scheme 8:**
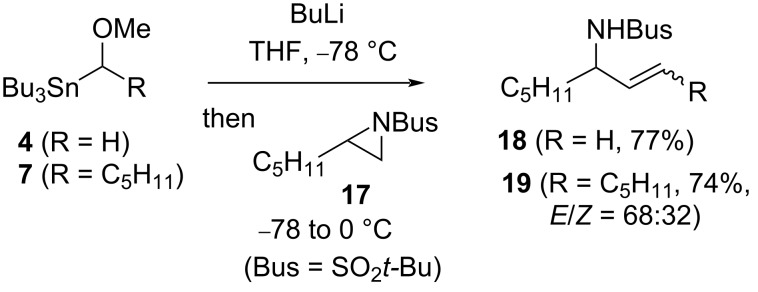
Allylic amines from aziridine **17**.

Analogous chemistry to that described above (Scheme 3 and Scheme 4) was found to be possible with a terminal aziridine **17**, providing access to the corresponding *N*-Bus-protected allylic amines **18** [19] and **19** (Scheme 8). In these cases, the amines are formed by preferential β-elimination [20–21] of lithium methoxide rather than BusNLi_2_.

**Scheme 9:**
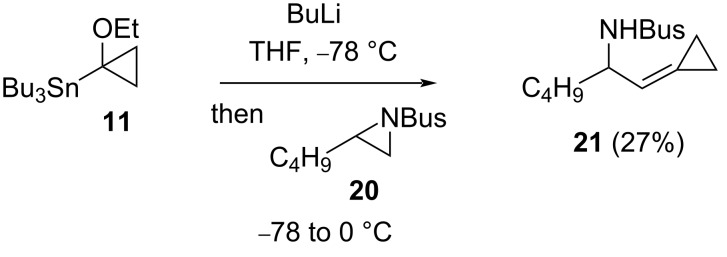
Cyclopropylidene synthesis from aziridine **20**.

Synthesis of cyclopropylidene **21** (Scheme 9), suggests a terminal *N*-Bus-aziridine is capable of being deprotonated by the α-lithio cyclopropane from stannane **11**; this contrasts with cross-coupling using the same carbenoid and epoxide **5** (Scheme 5), where the presence of LTMP also proved necessary.

**Scheme 10:**
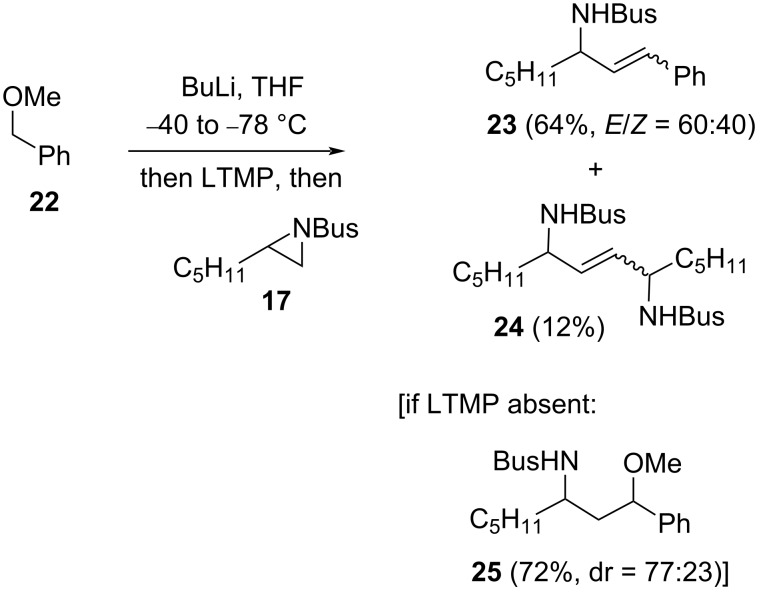
Cinnamylamine **23** synthesis from aziridine **17**.

A cinnamylamine **23** could be obtained in a tin-free process (Scheme 10), which utilises the increased acidity of a benzylic ether **22**. In this case, the presence of LTMP was necessary as only γ-amino ether **25** was observed in its absence. It was also important to carry out the reaction at −78 °C to avoid a 1,2-Wittig rearrangement of the lithiated benzyl ether [22]; this restricts the reaction to *N-*Bus-aziridines, as epoxides are not deprotonated by LTMP at such low temperatures. Alongside the cinnamylamine **23**, small amounts of the aziridine-derived carbenoid dimerisation product, 2-ene-1,4-diamine **24** [5], were observed. While the reaction profile was not altered on a solvent switch to hexane (**23** (62%, *E*/*Z* = 61:39); **24** (16%)), the yield of cinnamylamine **23** was slightly improved in hexane (69%, *E*/*Z* = 62:38) and the amount of dimer **24** curtailed (8%) by reducing the amount of LTMP from 2 to 1.2 equiv.

**Scheme 11:**
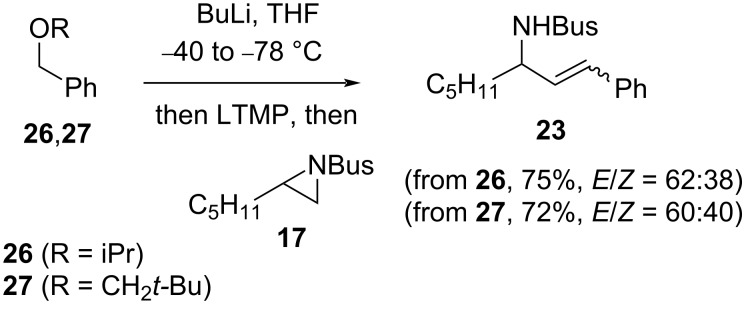
Cinnamylamine **23** synthesis from isopropyl or neopentyl benzylic ethers **26** and **27**.

The viability of a benzyl ether (Scheme 10) in the carbenoid eliminative cross-coupling offered a straightforward way to probe any effect of the size of the leaving group on stereoselectivity. However, neither isopropyl or neopentyl benzylic ethers **26** and **27** [23–24] led to a significant change in the *E*/*Z* ratio for cinnamylamine **23** (Scheme 11).

## Conclusion

In summary, we report a new, convergent access to allylic alcohols and amines. The process proceeds by selective cross-coupling of α-lithio terminal epoxides or *N*-Bus-aziridines with α-lithio ethers. Where 1,2-disubstituted alkenes are generated the *E*/*Z* stereoselectivity is modest, and preliminary results suggest the size of the leaving group does not play a significant role. However, the geometry of alkene formation might be controllable by using enantiomerically pure coupling partners [2]. Such terminal epoxides and aziridines are readily available [3,5], while the corresponding α-lithio ethers can be accessed from enantioenriched α-stannyl ethers [25]. The enantiopure variants await future investigation.

## Supporting Information

File 1 Experimental procedures and characterisation data for all new compounds.
