# Supplementary material for: Allylic alcohols and amines by carbenoid eliminative cross-coupling using epoxides or aziridines
Source: Beilstein J Org Chem. 2021 Sep 10;17:2385–9. doi: 10.3762/bjoc.17.155 (PMC8450947; doi:10.3762/bjoc.17.155)
Supplement: File 1 — Experimental procedures and characterisation data for all new compounds. [file Beilstein_J_Org_Chem-17-2385-s001.pdf]

BEILSTEIN JOURNAL OF ORGANIC CHEMISTRY

Supporting Information

for

Allylic alcohols and amines by carbenoid eliminative cross-coupling using epoxides or aziridines

Matthew J. Fleming and David M. Hodgson

Beilstein J. Org. Chem. **2021,** *17,* 2385–2389. doi:10.3762/bjoc.17.155

Experimental procedures and characterisation data for all new compounds

License and Terms: This is a supporting information file under the terms of the Creative Commons Attribution License (https://creativecommons.org/licenses/by/4.0). Please note that the reuse, redistribution and reproduction in particular requires that the author(s) and source are credited and that individual graphics may be subject to special legal provisions.
The license is subject to the *Beilstein Journal of Organic Chemistry* terms and conditions: (https://www.beilstein-journals.org/bjoc/terms)

General

All reactions requiring anhydrous conditions were conducted in flame-dried glassware under an atmosphere of argon. All solvents were either distilled under an atmosphere of argon (ethers from sodium benzophenone ketyl, hydrocarbons from CaH_2), or were degassed and dried over alumina under nitrogen [1]. Other reagents were used as obtained from commercial sources or purified in accordance with standard recommendations. Petrol refers to the fraction of light petroleum boiling at 30–40 °C.

Reactions were monitored by thin layer chromatography using commercially available aluminium-backed plates pre-coated with silica containing a fluorescent indicator (0.2 mm, Merck 60 F_{254}). Visualisation of reaction components was achieved with 354 nm light where possible and/or with phosphomolybdic acid or potassium permanganate. Column chromatography was performed on silica gel (Kieselgel 60, 40–3 μm).

Melting points (mp) were determined using a Kofler hot block apparatus and are uncorrected. Infrared spectra were recorded as thin films (neat or $CHCl_3$ solutions) on NaCl discs, or as KBr discs and using a 1750 FTIR spectrophotometer. Peak intensities are specified as strong (s), medium (m), weak (w) or broad (br). Only selected absorbencies are reported. ^1H and ^{13}C NMR spectra were recorded at 25 °C, with DPX400 or AV400 spectrometers. ^1H and ^{13}C NMR spectra were recorded in $CDCl_3$. Data are expressed as chemical shifts in parts per million, relative to residual $CHCl_3$ (^1H δ 7.27) or $CDCl_3$ (^{13}C δ 77.0). The multiplicity of each signal is designated by the following abbreviations: s, singlet; d, doublet; t, triplet; q, quartet; quint, quintet; sept, septet; br, broad singlet. Coupling constants *J* are given in Hz. GCMS analyses were performed on a Micromass GCT spectrometer with BPX5 column - HP 6890 (dimethylsilicone capillary column, 30 m, 0.25 mm internal diameter) equipped with a mass selective detector operating at 60 eV (CI). Flow rate (He) = 1 mL/min. Mass spectra were obtained from the EPSRC National Mass Spectrometry Service Centre, University of Wales, Swansea with a Micromass ZAB-E instrument, or 900 XLT high resolution double focusing mass spectrometer with tandem ion trap. Alternatively, they were recorded in-house using a VG Mass Lab TRIO1 or Micromass platform APCI spectrometer using chemical ionization (CI) or electron ionization (EI) techniques.

Solutions of commercially available organolithiums were titrated prior to use by adding a solution of the organometallic to a known concentration of 2,2,2-trimethylpropionanilide (in THF) until a colour change occurred [2].

General procedure: Allylic alcohol or *N*-Bus allylic amine synthesis by the addition of *α*-alkoxy organolithiums to epoxides or *N*-Bus aziridines.

To a solution of the appropriate stannane (3 equiv) in THF at −78 °C was added *n*-BuLi (3 equiv) dropwise. The reaction mixture was left at this temperature for 10 min and then the appropriate epoxide or aziridine (1 equiv) was added. The reaction mixture was allowed to warm to 0 °C over 4 h and then left to stir at this temperature for 2 h. After quenching with sat. brine solution, the layers were separated. The aqueous layer was extracted with Et_2O (× 2) and the combined organic layers were dried ($MgSO_4$) and evaporated under reduced pressure. The residue was purified by column chromatography (Et_2O/petrol) to give the corresponding allylic alcohol or *N*-Bus allylic amine.

1-Tridecen-3-ol (**6**) (Scheme 3 conditions)

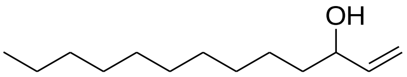

Following the general procedure, the addition of 1,2-epoxydodecane (**5**, 61 mg, 0.33 mmol) to (methoxymethyl)lithium [(generated from tributyl(methoxymethyl)stananne (**4** [3], 321 mg, 1.0 mmol) and *n*BuLi (1.6 M in hexanes; 0.63 mL, 1.0 mmol)] in THF (5 mL) gave after workup and column chromatography (10% Et_2O in petrol) allylic alcohol **6** [4] (52 mg, 79%) as a colourless oil; R_f 0.13 (10% Et_2O in petrol); IR (neat)/cm^{-1} 3355s, 1645m, 1465w, 990s; ^1H NMR (400 MHz) δ 5.92−5.83 (m, 1H), 5.16 (dq, *J* = 17, 1.5, 1H), 5.11 (dq, *J* = 10.5, 1.5, 1H), 4.10 (q, *J* = 6, 1H), 1.60 (br, 1 H), 1.55−1.21 (m, 18 H), 0.88 (t, *J* = 6.5, 3H); ^{13}C NMR (100 MHz) δ 141.3, 114.5, 73.3, 37.0, 31.9, 29.6, 29.5, 29.3, 25.3, 22.7, 14.1; MS *m*/*z* (CI) 216 ($M+NH_4^+$, 61), 198 (100), 180 (84), 81 (42); HRMS calcd for $C_{13}H_{30}NO$ ($M+NH_4^+$) 216.2327, found 216.2323.

6-Octadecen-8-ol (**8**) (Scheme 4 conditions)

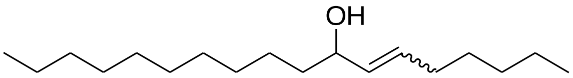

Following the general procedure, the addition of 1,2-epoxydodecane (**5**, 100 mg, 0.54 mmol) to (1-methoxyhexyl)lithium [(generated from tributyl-1-(methoxyhexyl)stananne (**7** [5,6], 660 mg, 1.63 mmol) and *n*-BuLi (1.6 M in hexanes; 1.02 mL, 1.63 mmol)] in THF (8 mL) gave after workup and column chromatography (10% Et_2O in petrol) allylic alcohol **8** (115 mg, 79%, *E:Z* = 73:27 by GCMS analysis, t_R *Z*-**8** 13.05 min, t_R *E*-**8** 13.18 min, initial temp. 80 °C, max. temp. 280 °C, rate 15 °C/min) as a colourless oil; R_f 0.13 (10% Et_2O in petrol); IR (neat)/cm^{-1} 3335br, 2925s, 2855s, 1466m, 1378m, 969m; ^{1}H NMR (400 MHz) δ 5.62 (dt, *J* = 15, 6.5, 0.7H), 4.01 (q, *J* = 6.5, 0.7H) (*E*-isomer), 5.40−5.28 (m, 0.3H), 4.41 (dt, *J* = 7, 6.5, 0.3H) (Z-isomer), 5.50−5.41 (m, 1H), 2.13−1.99 (m, 2H), 1.64−1.16 (m, 25H), 0.93−0.82 (m, 6H) (*E*- and *Z*-isomers); ^{13}C NMR (100 MHz) δ 133.1, 132.7, 132.2, 132.1, 73.2, 67.7, 37.5, 37.3, 32.14, 31.9, 31.5, 31.4, 29.6, 29.6, 29.4, 29.3, 28.9, 27.7, 25.5, 25.4, 22.7, 22.5, 14.1, 14.0, 14.0 (*E*- and *Z*-isomers); MS *m*/*z* (CI) 286 (M+NH_4^+, 18), 269 (30), 268 (100); HRMS calcd for $C_{18}H_{40}NO$ (M+NH_4^+) 286.3104, found 286.3104.

1-Hept-1-enyl-cyclododecanol (**10**) (Scheme 4 conditions)

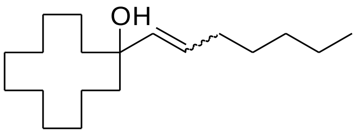

Following the general procedure, the addition of 1-oxa-spiro[2.11]tetradecane (**9** [7], 100 mg, 0.54 mmol) to (1-methoxyhexyl)lithium [(generated from tributyl-1-(methoxyhexyl)stananne (**7** [5,6], 660 mg, 1.63 mmol) and *n*BuLi (1.6 M in hexanes; 1.02 mL, 1.63 mmol)] in THF (8 mL) gave after workup and column chromatography (10% Et_2O in petrol) allylic alcohol **10** (*E:Z* = 82:18 by GCMS analysis of the crude reaction mixture, t_R *Z*-**10** 14.68 min, t_R *E*-**10** 14.70 min, initial temp. 80 °C, max. temp. 280 °C, rate 15 °C/min). First to elute was *Z*-**10** (21 mg, 13%) as a white solid; R_f 0.40 (10% Et_2O in petrol); mp 51−54 °C; IR (neat)/cm^{-1} 3329br, 2926s, 1601w, 1470m, 1161w; ^{1}H NMR (400 MHz) δ 5.40−5.31 (m, 2H), 2.37 (q, *J* = 6.5, 2H), 1.71−1.50 (m, 4H), 1.43−1.23 (m, 25H), 0.90 (t, *J* = 7, 3H); ^{13}C NMR (100 MHz) δ 135.3, 132.1, 76.0, 36.1, 31.6, 29.8, 28.5, 26.4, 26.0, 22.6, 22.2, 19.7, 14.0; MS *m*/*z* (CI) 280 (M+H$^+$, 30), 264 (32), 262 (100); HRMS calcd for $C_{19}H_{36}O$ (M+H$^+$)

280.2761, found 280.2758. Second to elute was *E*-**10** (94 mg, 59%) as a colourless oil; R_f 0.13 (10% Et_2O in petrol); IR (neat)/cm^{-1} 3382s, 2928s, 1470m, 1347m, 973m; ^{1}H NMR (400 MHz) δ 5.66–5.54 (m, 2H), 2.02 (q, *J* = 7, 2H), 1.70–1.57 (m, 4H), 1.52–1.19 (m, 25H), 0.89 (t, *J* = 7, 3H); ^{13}C NMR (100 MHz) δ 137.2, 127.7, 74.9, 35.1, 32.4, 31.4, 29.1, 26.5, 26.0, 22.6, 22.5, 22.3, 22.2, 19.7, 14.0; MS *m*/*z* (CI) 280 (M+H$^+$, 18), 264 (28), 263 (100); HRMS calcd for $C_{19}H_{36}O$ (M+H$^+$) 280.2761, found 280.2763.

1-Cyclopropylidenedodecan-2-ol (**12**) (Scheme 5 conditions)

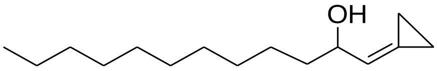

To a solution of tributyl(1-ethoxycyclopropyl)stannane (**11** [8], 198 mg, 0.52 mmol) and 2,2,6,6-tetramethylpiperidine (73 mg, 0.52 mmol) in THF (4.5 mL) at −78 °C was added *n*-BuLi (1.6 M in hexanes; 0.65 mL, 1.04 mmol) dropwise. After 15 min 1,2-epoxydodecane (**5**, 48 mg, 0.26 mmol) was added and the reaction was allowed to warm to 0 °C over 4 h and left to stir at this temperature for 2 h. After quenching with sat. brine solution (10 mL), the layers were separated. The aqueous layer was extracted with Et_2O (2 × 10 mL), the combined organic layers were dried ($MgSO_4$) and evaporated under reduced pressure. The residue was purified by column chromatography (10% Et_2O in petrol) to give allylic alcohol **12** (18 mg, 30%) as a colourless oil; R_f 0.10 (10% Et_2O in petrol); IR (neat)/cm^{-1} 3346br, 3054w, 2925s, 2854s, 1466m, 1007; ^{1}H NMR (400 MHz) δ 5.84 (dquint, *J* = 7, 2, 1H), 4.30 (quint, *J* = 7, 1H), 1.69 (br, 1H), 1.66–1.54 (m, 2H), 1.41–1.26 (m, 16H), 1.18–1.04 (m, 4H), 0.88 (t, *J* = 7, 3H); ^{13}C NMR (100 MHz) δ 122.9, 121.4, 72.7, 37.3, 31.9, 29.6, 29.3, 25.4, 22.7, 14.1, 2.0, 1.4; MS *m*/*z* (CI) 242 (M+ NH_4^+, 56), 225 (100), 224 (47), 207 (28), 195 (12); HRMS calcd for $C_{15}H_{32}NO$ (M+NH_4^+) 242.2484, found 242.2491.

(*E*)- and (*Z*)-1-Trimethylsilanyl-1-tridecen-3-ol (**14**) (Scheme 6 conditions)

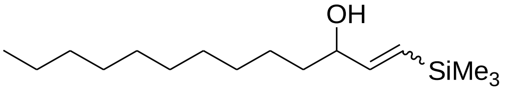

To a solution of (methoxymethyl)trimethylsilane (**13**, 0.13 mL, 0.81 mmol) in THF (0.8 mL) at −78 °C was added *s*BuLi (1.4 M in hexanes; 0.58 mL, 0.81 mmol) dropwise. The reaction mixture was warmed to −35 °C and stirred at this temperature for 1 h and then warmed to

0 °C. A solution of LTMP (prepared by the addition of *n*BuLi (1.6 M in hexanes; 0.54 mL, 0.81 mmol) to 2,2,6,6-tetramethylpiperidine (115 mg, 0.81 mmol) in hexane (7 mL) at 0 °C) was added dropwise. The reaction was left for a further 5 min at 0 °C and then 1,2-epoxydodecane (**5**, 100 mg, 0.54 mmol) was added. The reaction mixture was stirred for a further 2 h at 0 °C. After quenching with sat. brine solution (10 mL), the layers were separated. The aqueous layer was extracted with Et_2O (2 × 10 mL), the combined organic layers were dried ($MgSO_4$), and evaporated under reduced pressure. The residue was purified by column chromatography (10% Et_2O in petrol) to give vinylsilane **14** (38 mg, 26%, *E*:*Z* = 81:19 by ^{1}H NMR analysis of vinylic protons in the δ 6.50−6.00 region) as a colourless oil; R_f 0.16 (10% Et_2O in petrol); IR (neat)/cm^{-1}; 3334br, 2925s, 2855s, 1620w, 1466m, 1248s, 838s; ^{1}H NMR (400 MHz) δ 6.04 (dd, *J* = 18.5, 5.5, 0.8H), 5.84 (d, *J* = 18.5, 0.8H), 4.07 (dt, *J* = 6, 5.5, 0.8H), 0.08 (s, 7.2H) (*E*-isomer) 6.23 (dd, *J* = 14, 9 ,0.2H), 5.65 (d, *J* = 14, 0.2H), 4.22 (dt, *J* = 9, 6.5, 0.2H), 0.15 (s, 1.8H) (*Z*-isomer) 1.64−1.23 (m, 17H), 0.89 (t, *J* = 7, 3H) (*E*- and *Z*-isomer); ^{13}C NMR (100 MHz) δ 150.2, 148.7, 131.7, 129.1, 74.7, 72.4, 37.2, 36.9, 31.9, 29.6, 29.6, 29.6, 29.5, 29.3, 25.4, 25.4, 22.7, 14.1, 0.43, -1.32 (*E*- and *Z*- isomers); MS *m*/*z* (CI) 270 ($M+NH_4^+$, 25), 90 (100); HRMS calcd for $C_{16}H_{38}ONSi$ ($M+NH_4^+$) 288.2717, found 288.2716. Also isolated was allylic alcohol **6** (21 mg, 20%); data as above.

6-Octadecen-8-ol (**8**) from phenyl hexyl sulfone (Scheme 7 conditions)

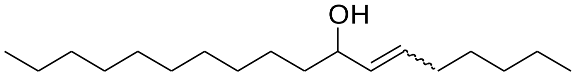

To a solution of phenyl hexyl sulfone (**15** [9], 148 mg, 0.65 mmol) in THF (0.7 mL) at −78 °C was added *n*-BuLi (2.5 M in hexanes; 0.26 mL, 0.65 mmol) dropwise. After 15 min a solution of LTMP (prepared by the addition of *n*-BuLi (2.5 M in hexanes; 0.43 mL, 1.09 mmol) to 2,2,6,6-tetramethylpiperidine (150 mg, 1.09 mmol) in hexane (7 mL) at 0 °C) was added dropwise. The reaction was warmed to 0 °C, 1,2-epoxydodecane (**5**, 100 mg, 0.54 mmol) added and the reaction left stirring for 2 h. After quenching with sat. brine solution (10 mL), the layers were separated. The aqueous layer was extracted with Et_2O (2 × 10 mL), the combined organic layers were dried ($MgSO_4$) and evaporated under reduced pressure. The residue was purified by column chromatography (10% Et_2O in petrol → 40%

Et_2O in petrol) to give allylic alcohol **8** (80 mg, 55%, *E:Z* = 56:44 by GCMS analysis, t_R *Z*-**8** isomer 12.47 min, , t_R *E*-**8** 12.60 min, initial temp. 80 °C, max. temp. 280 °C, rate 15 °C /min) as a colourless oil. R_f 0.29 (20% Et_2O in petrol). Other data as above. Also isolated was 6-benzenesulfonyl-octadecan-8-ol (**16**) (69 mg, 44%, dr = 50:50 by ^{1}H NMR analysis, diastereoisomers separated by chromatography). Data for the faster eluting diastereoisomer: a colourless oil; R_f 0.28 (40% Et_2O in petrol); IR (neat)/cm^{-1} 3521br, 3066w, 2925s, 1586w, 1447s, 1301s, 1144s, 1084s; ^{1}H NMR (400 MHz) δ 7.92 (m, 2H), 7.69–7.65 (m, 1H), 7.60–7.56 (m, 2H), 3.92–3.88 (m, 1H), 3.34–3.28 (m, 1H), 2.17 (br, 1H), 2.02 (ddd, *J* = 12. 8.5, 3.5, 1H), 1.79–1.62 (m, 3H), 1.47–1.11 (m, 24H), 0.89 (t, *J* = 7, 3H), 0.83 (t, *J* = 7, 3H); ^{13}C NMR (100 MHz) δ 137.8, 133.6, 129.1, 128.8, 68.9, 61.2, 37.9, 35.8, 31.9, 31.3, 29.7, 29.6, 29.6, 29.4, 29.3, 25.9, 25.6, 22.7, 22.2, 14.1, 13.9; MS *m/z* (CI) 411 (M+H$^+$, 21), 268 (32), 267 (100), 256 (37), 126 (88), 110 (69); HRMS calcd for $C_{14}H_{43}O_3S$ (M+H$^+$) 411.2933, found 411.2944. Data for the slower eluting diastereoisomer: a colourless oil; R_f 0.17 (40% Et_2O in petrol); IR (neat)/cm^{-1} 3508br, 3066w, 2925s, 1586w, 1447s, 1302s, 1145s; ^{1}H NMR (400 MHz) δ 7.91–7.89 (m, 2H), 7.68–7.65 (m, 1H), 7.59–7.56 (m, 2H), 3.66–3.62 (m, 1H), 3.31–3.25 (m, 1H), 2.18 (br, 1H), 1.97 (ddd, *J* = 14.5, 10, 4.5, 1H), 1.85–1.69 (m, 3H), 1.59–1.15 (m, 24H), 0.89 (t, *J* = 7, 3H), 0.84 (t, *J* = 7, 3H); ^{13}C NMR (100 MHz) δ 137.8, 133.6, 129.1, 128.9, 69.5, 61.9, 38.4, 35.4, 31.9, 31.6, 29.6, 29.5, 29.3, 28.7, 26.2, 25.5, 22.7, 22.2, 14.1, 13.9; MS *m/z* (CI) 411 (M+H$^+$, 41), 393 (25), 267 (100), 126 (55), 125 (60), 110 (78), 95 (45); HRMS calcd for $C_{14}H_{43}O_3S$ (M+H$^+$) 411.2933, found 411.2938.

2-Methyl-*N*-(1-octen-3-yl)propane-2-sulfonamide (**18**) (Scheme 8 conditions)

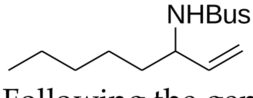

Following the general procedure, addition of 1-(*tert*-butylsulfonyl)-2-pentylaziridine (**17** [10,11], 78 mg, 0.33 mmol) to (methoxymethyl)lithium [(generated from tributyl(methoxymethyl)stananne (**4** [3], 321 mg, 1.0 mmol) and *n*-BuLi (1.6 M in hexanes; 0.63 mL, 1.0 mmol)] in THF (5 mL) gave after workup and column chromatography (30% Et_2O in petrol) allylic sulfonamide **18** [12] (63 mg, 77%) as a colourless oil; R_f 0.21 (30% Et_2O in petrol); IR (neat)/cm^{-1} 3281br, 3083w, 2933s, 1645w, 1455m, 1304s, 1126s; ^{1}H NMR (400 MHz) δ 5.77 (ddd, *J* = 17, 10.5, 6.5, 1H), 5.21 (ddd, *J* = 17, 1.5, 1, 1H), 5.13 (ddd, *J* = 10.5,

1.5, 1, 1H), 4.24 (d, J = 9.5, 1H), 3.92−3.84 (m, 1H), 1.63−1.50 (m, 2H), 1.45−1.21 (m, 15H), 0.86 (t, J = 7, 3H); ^{13}C NMR (100 MHz) δ 138.9, 115.2, 59.6, 57.3, 37.0, 31.5, 25.1, 24.2, 22.4, 13.9; MS m/z (CI) 265 (M+NH$_4^+$, 100), 248 (28), 176 (21), 128 (79), 111 (42), 56 (43); HRMS calcd for $C_{12}H_{29}N_2O_2S$ (M+NH$_4^+$) 265.1950, found 265.1958.

(*E*)- and (*Z*)-2-Methyl-*N*-(7-tridecen-6-yl)propane-2-sulfonamide (**19**) (Scheme 8 conditions)

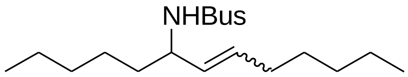

Following the general procedure, addition of 1-(*tert*-butylsulfonyl)-2-pentylaziridine (**17** [10,11], 127 mg, 0.54 mmol) to (1-methoxyhexyl)lithium [(generated from tributyl-1-(methoxyhexyl)stananne (**7** [5,6], 660 mg, 1.63 mmol) and *n*BuLi (1.6 M in hexanes; 1.02 mL, 1.63 mmol)] in THF (8 mL) gave after workup and column chromatography (10%→20% Et_2O in petrol) allylic sulfonamide **19** (*E:Z* = 68:32 by GCMS analysis of the crude reaction mixture, t_R *E*-**19** 11.38 min, t_R *Z*-**19** 11.52 min, initial temp. 80 °C, max. temp. 280 °C, rate 20 °C/min) First to elute was *E*-**19** (86 mg, 50%) as a colourless oil; R_f 0.16 (20% Et_2O in petrol); IR (neat)/cm^{-1} 3278br, 2929s, 1601w, 1456m, 1306s, 1128s; ^{1}H NMR (400 MHz) δ 5.60 (dt, J = 15.5, 7, 1H), 5.33 (dd, J = 15.5, 7, 1H), 3.90−3.79 (m, 2H), 1.62−1.19 (m, 14 H), 0.88 (t, J = 7, 6H); ^{13}C NMR (100 MHz) δ 132.2, 130.6, 59.6, 56.9, 37.9, 32.2, 31.5, 31.3, 28.8, 25.3, 24.3, 22.5, 22.5, 14.0, 14.0; MS m/z (CI) 335 (M+NH$_4^+$, 9), 246 (48), 198 (52), 181 (62), 126 (100); HRMS calcd for $C_{17}H_{39}NOS$ (M+NH$_4^+$) 335.2732, found 335.2731. Second to elute was *Z*-**19** (41 mg, 24%) as a colourless oil; R_f 0.13 (20% Et_2O in petrol); IR (neat)/cm^{-1} 3276br, 2929s, 1600w, 1457s, 1366m, 1306s, 1209w, 1127s; ^{1}H NMR (400 MHz) δ 5.46 (dt, J = 10, 7, 1H), 5.20 (ddd, J = 10, 9, 1.5, 1H), 4.21 (tt, J = 9, 7, 1H), 3.73 (d, J = 9, 1H), 2.05 (dtd, J = 7, 6, 1.5, 2H), 1.68−1.59 (m, 2H), 1.49−1.23 (m, 12H), 0.89 (t, J = 6.5, 6H); ^{13}C NMR (100 MHz) δ 131.7, 130.4, 59.5, 52.3, 38.0, 31.6, 31.5, 29.1, 27.7, 25.3, 24.2, 22.5, 22.5, 14.0, 14.0; MS m/z (CI) 335 (M+NH$_4^+$, 15), 246 (41), 198 (41), 181 (49), 126 (100); HRMS calcd for $C_{17}H_{39}NOS$ (M+NH$_4^+$) 335.2732, found 335.2728.

N-(1-Cyclopropylidenehexan-2-yl)-2-methylpropane-2-sulfonamide (**21**) (Scheme 9 conditions)

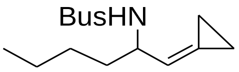

Following the general procedure, addition of 1-(*tert*-butylsulfonyl)-2-butylaziridine (**20** [13], 37 mg, 0.17 mmol) to 1-(ethoxycyclopropyl)lithium [(generated from tributyl(1-ethoxycyclopropyl)stannane (**13** [8], 198 mg, 0.52 mmol) and *n*-BuLi (1.6 M in hexanes; 0.33 mL, 0.52 mmol)] in THF (4 mL) gave after workup and column chromatography (20% Et_2O in petrol) allylic sulfonamide **21** (12 mg, 27%) as a colourless oil; R_f 0.12 (20% Et_2O in petrol); IR (neat)/cm^{-1} 3279br, 2933s, 1457m, 1306s, 1127s; ^{1}H NMR (400 MHz) δ 5.79 (dquint, *J* = 7, 2, 1H), 4.10 (dquint, *J* = 9, 7, 1H), 3.93 (d, *J* = 9, 1H), 1.72−1.54 (m, 4H), 1.45−1.26 (m, 11H), 1.19−1.06 (m, 4H), 0.90 (t, *J* = 7, 3H); ^{13}C NMR (100 MHz) δ 123.3, 119.3, 59.6, 56.7, 37.2, 27.7, 24.2, 22.5, 14.0, 2.8, 1.5; MS *m*/*z* (CI) 277 ($M+NH_4^+$, 53), 260 (100); HRMS calcd for $C_{13}H_{29}NO_2S$ ($M+NH_4^+$) 277.1950, found 277.1948.

(*E*)- and (*Z*)-2-Methylpropane-2-sulfonic acid(1-pentyl-3-phenylallyl)amide (**23**) (Scheme 11 conditions)

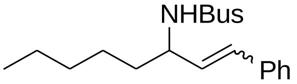

To a solution of benzyl isopropyl ether (**26** [14], 103 mg, 0.69 mmol) in THF (1.5 mL) at −40 °C was added *n*-BuLi (2.5 M in hexanes; 0.28 mL, 0.69 mmol) dropwise. After 1 h the reaction was cooled to −78 °C and a solution of LTMP (prepared by the addition of *n*-BuLi (2.5 M in hexanes; 0.17 mL, 0.42 mmol) to 2,2,6,6-tetramethylpiperidine (58 mg, 0.42 mmol) in hexane (4 mL) at 0 °C) was added dropwise. 1-(*tert*-Butylsulfonyl)-2-pentylaziridine (**17** [10,11], 81 mg, 0.35 mmol) in hexane (0.5 mL) was added and the reaction was left for 2 h at −78 °C. After quenching with sat. aq. NH_4Cl solution (10 mL), the layers were separated. The aqueous layer was extracted with Et_2O (2 × 10 mL), the combined organic layers were dried ($MgSO_4$), and evaporated under reduced pressure. The residue was purified by column chromatography (15% Et_2O in petrol) to give allylic sulfonamide **23** (85 mg, 75%, *E*:*Z* = 62:38 by GCMS analysis; t_R *Z*-**23** 14.58 min, t_R *E*-**23** 15.37 min, initial temp. 80 °C, max. temp. 280 °C, rate = 15 °C/min) as a colourless oil; R_f 0.08 (15% Et_2O in petrol); IR

(neat)/cm^{-1} 3277br, 2931s, 2859s, 1600w, 1453m, 1365s, 1127s; ^{1}H NMR (400 MHz) δ, 6.56 (d, *J* = 16, 0.6H), 6.11 (dd, *J* = 16, 7, 0.6H), 4.11 (dquint, *J* = 9.5, 7, 0.6H), 3.96 (d, *J* = 9.5, 0.6H), (*E*-isomer); 6.50 (d, *J* = 11.5, 0.4H), 5.53 (dd, *J* = 11.5, 10, 0.4H), 4.52 (ddt, *J* = 10, 8.5, 7, 0.4H), 3.88 (d, *J* = 8.5 0.4H) (*Z*-isomer) 7.43−7.21 (m, 5H), 1.75−1.56 (m, 4H), 1.48−1.21 (m, 13H), 0.90 (t, *J* = 6.8, 3H) (*E*- and *Z*-isomer); ^{13}C NMR (100 MHz) δ 136.5, 136.4, 133.1, 130.8, 130.3, 129.7, 128.6, 128.4, 128.4, 127.7, 127.2, 126.4, 59.7, 59.5, 57.0, 52.6, 37.8, 37.5, 31.5, 31.5, 25.3, 25.1, 24.3, 24.1, 22.5, 22.3, 14.0, 14.0 (*E*- and *Z*- isomers); MS *m*/*z* (EI+) 323 (M^+, 5), 252 (26), 202 (24), 186 (53), 143 (58), 132 (88), 130 (51), 129 (100), 128 (80), 115 (47); HRMS calcd for $C_{18}H_{29}NO_2S$ (M^+) 323.1919, found 323.1928.

(When ene-disulfonamide **24** was observed, it was identified by comparison to our lit. data [11].)

2-Methyl-propane-2-sulfonic acid [1-(2-methoxy-2-phenylethyl)hexyl]amide (**25**) (Scheme 10 conditions)

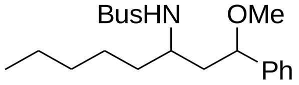

To a solution of benzyl methyl ether (**22**, 128 mg, 1.05 mmol) in THF (5 mL) at −40 °C was added *n*-BuLi (2.5 M in hexanes; 0.42 mL, 1.05 mmol) dropwise. After 1 h the reaction was cooled to −78 °C and 1-(*tert*-butylsulfonyl)-2-pentylaziridine (**17** [10,11], 81 mg, 0.35 mmol) in THF (0.5 mL) was added and the reaction was left for 2 h. After quenching with sat. brine solution (10 mL), the layers were separated. The aqueous layer was extracted with Et_2O (2 × 10 mL), the combined organic layers were dried ($MgSO_4$), and evaporated under reduced pressure. The residue was purified by column chromatography (20% Et_2O in petrol) to give sulfonamide **25** (81 mg, 72%, dr = 77:23 by ^{1}H NMR analysis of isomeric benzylic protons in the δ 4.50−4.00 region) as a white solid; R_f 0.11 (20% Et_2O in petrol); IR (neat)/cm^{-1} 3279br, 2932s, 1455m, 1303s, 1126s; ^{1}H NMR (400 MHz) δ 7.41−7.38 (m, 5H), 4.45 (d, *J* = 7.5, 1H), 4.30 (dd, *J* = 9, 3.5, 1H), 3.75−3.66 (m, 1H), 3.19 (s, 3H), 1.98 (ddd, *J* = 14.5, 9, 7.5, 1H), 1.81 (ddd, *J* = 9.5, 6, 3.5, 1H), 1.62−1.56 (m, 2H), 1.44−1.21 (m, 15H), 0.88 (t, *J* = 7, 3H) (major diastereoisomer); 4.81 (d, *J* = 9.5, 0.2H), 4.43−4.41 (m, 0.2H), 3.65−3.56 (m, 0.2H) (identification of minor diastereoisomer) ^{13}C NMR (100 MHz) δ 141.8, 128.6, 127.8,

126.5, 82.0, 59.3, 56.4, 53.1, 43.3, 36.3, 31.7, 24.7, 24.4, 24.3, 22.5 (major diastereoisomer); 141.7, 128.6, 127.8, 126.4, 80.8, 59.5, 56.4, 53.1, 43.2, 36.0, 31.7, 24.7, 24.4, 24.3, 22.6, 14.0 (minor diastereoisomer); MS *m/z* (CI) 356 ($M+H^+$, 7), 237 (56), 220 (76), 187 (22), 135 (21), 121 (43), 100 (100); HRMS calcd for $C_{19}H_{34}NO_3S$ ($M+NH_4^+$) 356.2259, found 356.2260.

Benzyl neopentyl ether (**27**)

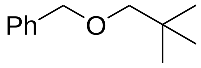

Neopentyl alcohol (1.0 mL, 9.26 mmol) was added to a suspension of NaH (60% w/w, 404 mg, 10.1 mmol) in THF (10 mL) and stirred for 30 min. TBAI (40 mg, 1.07 mmol) and then benzyl bromide (1.0 mL, 8.4 mmol) were added and the reaction stirred for 14 h. After quenching with sat. brine solution (20 mL), the layers were separated. The aqueous layer was extracted with Et_2O (2 × 20 mL), the combined organic layers were dried ($MgSO_4$), and evaporated under reduced pressure. The residue was purified by column chromatography (100% petrol→5% Et_2O in petrol) to give ether **27** [15] (1.15 g, 80%) as a colourless oil; R_f 0.60 (5% Et_2O in petrol); IR (neat)/cm^{-1} 3031w, 2955s, 2866s, 1480s, 1385s, 1101s; ^{1}H NMR (400 MHz) δ 7.42–7.37 (m, 4H), 7.33–7.28 (m, 1H), 4.57 (s, 2H), 3.17 (s, 2H), 0.99 (s, 9H); ^{13}C NMR (100 MHz) δ 139.1, 128.2, 127.2, 127.2, 80.9, 73.2, 32.1, 26.8; MS *m/z* (CI) 196 ($M+NH_4^+$, 100), 179 (69), 108 (94), 91 (82); HRMS calcd for $C_{12}H_{22}NO$ ($M+NH_4^+$) 196.1701, found 196.1701.

References

1. Pangborn, A. B.; Giardello, M. A.; Grubbs, R. H.; Rosen, R. K.; Timmers, F. J. *Organometallics* **1996**, *15*, 1518−1520.

2. Suffert, J. *J. Org. Chem.* **1989**, *54*, 509−510.

3. Kaufman, T. S. *Synlett* **1997**, 1377−1378.

4. Yazbak, A.; Sinha, S. C.; Keinan, E. *J. Org. Chem.* **1998**, *63*, 5863−5868.

5. Still, W. C. *J. Am. Chem. Soc.* **1978**, *100*, 1481−1486.

6. Linderman, R. J.; Siedlecki, J. M. *J. Org. Chem.* **1996**, *61*, 6492−6493.

7. Michnick, T. J.; Matteson, D. S. *Synlett* **1991**, 631−632.

8. Gadwood, R. C.; Rubino, M. R.; Nagarajan, S. C.; Michel, S. T. *J. Org. Chem*. **1985**, *50*, 3255−3260.

9. Orita, A.; Yoshioka, N.; Struwe, P.; Braier, A.; Beckmann, A.; Otera, J. *Chem. - Eur. J.* **1999**, *5*, 1355−1363.

10. Hodgson, D. M.; Humphreys, P. G.; Ward, J. G. *Org. Lett.* **2005**, *7*, 1153−1156.

11. Hodgson, D. M.; Humphreys, P. G.; Miles, S. M.; Brierley, C. A. J.; Ward, J. G. *J. Org. Chem*. **2007**, *72*, 10009–10021.

12. Hodgson, D. M.; Fleming, M. J.; Stanway, S. J. *Org. Lett.* **2005**, *7*, 3295−3298.

13. Gontcharov, A. V.; Liu, H.; Sharpless, K. B. *Org. Lett.* **1999**, *1*, 783–786.

14. Kim, J. D.; Han, G.; Jeong, L. S.; Park, H.-J.; Zee, O. P.; Jung, Y. H. *Tetrahedron* **2002**, *58*, 4395−4402.

15. Caubere, P.; Moreau, J. *Tetrahedron* **1970**, *26*, 2637−2647.
